# Distinction of pseudoprogression from true progression in glioblastomas using machine learning based on multiparametric magnetic resonance imaging and O^6^-methylguanine-methyltransferase promoter methylation status

**DOI:** 10.1093/noajnl/vdae159

**Published:** 2024-10-03

**Authors:** Virendra Kumar Yadav, Suyash Mohan, Sumeet Agarwal, Laiz Laura de Godoy, Archith Rajan, MacLean P Nasrallah, Stephen J Bagley, Steven Brem, Laurie A Loevner, Harish Poptani, Anup Singh, Sanjeev Chawla

**Affiliations:** Centre for Biomedical Engineering, Indian Institute of Technology Delhi, New Delhi, India; Department of Radiology, Perelman School of Medicine at the University of Pennsylvania, Philadelphia, PA, USA; Yardi School of Artificial Intelligence, Indian Institute of Technology Delhi, New Delhi, India; Department of Electical Engineering, Indian Institute of Technology Delhi, New Delhi, India; Department of Radiology, Perelman School of Medicine at the University of Pennsylvania, Philadelphia, PA, USA; Department of Radiology, Perelman School of Medicine at the University of Pennsylvania, Philadelphia, PA, USA; Department of Clinical Pathology and Laboratory Medicine, Perelman School of Medicine at the University of Pennsylvania, Philadelphia, Pennsylvania, USA; Department of Medicine, Division of Hematology-Oncology, Perelman School of Medicine at the University of Pennsylvania, Philadelphia, Pennsylvania, USA; Department of Neurosurgery, Perelman School of Medicine at the University of Pennsylvania, Philadelphia, Pennsylvania, USA; Department of Radiology, Perelman School of Medicine at the University of Pennsylvania, Philadelphia, PA, USA; Department of Molecular and Clinical Cancer Medicine, Centre for Preclinical Imaging, University of Liverpool, Liverpool, UK; Centre for Biomedical Engineering, Indian Institute of Technology Delhi, New Delhi, India; Yardi School of Artificial Intelligence, Indian Institute of Technology Delhi, New Delhi, India; Department of Radiology, Perelman School of Medicine at the University of Pennsylvania, Philadelphia, PA, USA

**Keywords:** diffusion and perfusion MR imaging, glioblastoma, machine-learning, pseudoprogression, true progression

## Abstract

**Background:**

It is imperative to differentiate true progression (TP) from pseudoprogression (PsP) in glioblastomas (GBMs). We sought to investigate the potential of physiologically sensitive quantitative parameters derived from diffusion and perfusion magnetic resonance imaging (MRI), and molecular signature combined with machine learning in distinguishing TP from PsP in GBMs in the present study.

**Methods:**

GBM patients (*n* = 93) exhibiting contrast-enhancing lesions within 6 months after completion of standard treatment underwent 3T MRI. Final data analyses were performed on 75 patients as O^6^-methylguanine-DNA-methyltransferase (MGMT) status was available only from these patients. Subsequently, patients were classified as TP (*n* = 55) or PsP (*n* = 20) based on histological features or mRANO criteria. Quantitative parameters were computed from contrast-enhancing regions of neoplasms. PsP datasets were artificially augmented to achieve balanced class distribution in 2 groups (TP and PsP). A random forest algorithm was applied to select the optimized features. The data were randomly split into training and testing subsets in an 8:2 ratio. To develop a robust prediction model in distinguishing TP from PsP, several machine-learning classifiers were employed. The cross-validation and receiver operating characteristic (ROC) curve analyses were performed to determine the diagnostic performance.

**Results:**

The quadratic support vector machine was found to be the best classifier in distinguishing TP from PsP with a training accuracy of 91%, cross-validation accuracy of 86%, and testing accuracy of 85%. Additionally, ROC analysis revealed an accuracy of 85%, sensitivity of 70%, and specificity of 100%.

**Conclusions:**

Machine learning using quantitative multiparametric MRI may be a promising approach to distinguishing TP from PsP in GBMs.

## Lay Summary

Glioblastoma is a very aggressive type of brain cancer. After treatment with radiation and chemotherapy, it can be hard to know whether changes seen on an MRI scan are due to the treatment itself (pseudoprogression) or if the cancer is actually growing (progression). The authors of this study wanted to investigate if they could improve our ability to differentiate these 2 entities apart. To address this issue, they used advanced MRI techniques and genetic information from the tumors of 75 glioblastoma patients combined with several computational modeling programs. The authors found that they could accurately tell the difference between treatment effects and growth of cancer about 85%–90% of the time.

**Key Points**

- The current study demonstrated the potential of ML and quantitative MRI in differentiating TP from PsP in GBMs.
- SVM classifier with RBF kernel and quantitative MRI (DTI and DSC-PWI) provided the best results.
- FA, rCBV_max_, and MD were identified as key parameters in differentiating TP from PsP in GBMs.

**Importance of Study**

Identifying treatment “success” or ‘failure’ significantly influences clinical decision-making. As the management of GBM patients with TP and PsP is quite different, there is an unmet need to develop an objective and therapy-agnostic response assessment prediction model for providing real-time feedback to inform clinicians making therapeutic decisions, particularly in the early post-treatment window. Not only will this model help oncologists react quickly to early TP by switching therapies, or treat PsP conservatively, but more importantly it will help to relieve the uncertainty and stress associated with “scanxiety” as many patients and their loved ones experience a sense of dread and vulnerability.

Glioblastoma (GBM) is the most common and aggressive brain cancer.^1^ Despite being treated with maximal safe surgical resection followed by concurrent chemo-radiotherapy (CCRT) along with adjuvant temozolomide (TMZ).^1,2^ The vast majority of patients (~70%) present a new contrast-enhancing lesion on T_1_-weighted MR images in the radiation field within 6 months after the completion of CCRT.^3–5^ While this lesion may represent true tumor progression (TP), it may also reflect a predominance of treatment effect, colloquially termed as “pseudoprogression” (PsP) that is mediated by chemo-radiotherapy-induced increased vascular permeability leading to a profound inflammatory response in the treatment bed.^2^

The PsP is a transient phenomenon that usually stabilizes or resolves spontaneously without further treatment.^2^ A meta-analysis revealed that the incidence of PsP in GBMs treated with CCRT is approximately 36% (95% CI: 33%–40 %).^2^ This phenomenon is particularly more frequent in GBMs harboring promoter methylation of O^6^-methylguanine-methyltransferase (MGMT) and in those GBMs treated with novel immunotherapy regimens.^1,6^ In general, patients with PsP have better overall survival and are less likely to exhibit signs and symptoms of neurological deterioration than those with TP.^7,8^ Therefore, these patients are often (usually every 6–8 weeks) monitored with follow-up magnetic resonance imaging (MRI) scans. Moreover, these patients are symptomatically managed with the continuation of adjuvant TMZ without undergoing unnecessary second-stage surgery. On the other hand, patients with TP often require repeat biopsy/surgical resection and/or switching to alternative second-line therapies such as bevacizumab, tumor treating fields (TTFields), immunotherapy, or other clinical trials.^6,8,9^ Thus, identification of patients with PsP is critical to avoid unnecessary repeat surgeries and administration of expensive and potentially risky therapies.

On conventional neuroimaging both TP and PsP lesions exhibit similar features, thus presenting a considerable diagnostic dilemma to radiologists and oncologists.^8,10^ Some studies have documented the clinical utility of physiological imaging techniques such as diffusion and perfusion MRI in the assessment of treatment response in GBMs, with variable success rates.^11–16^ However, reported threshold values of a single or combination of diffusion and perfusion MRI-derived quantitative parameters vary widely across different studies, adversely impacting the investigator’s/physicians’ confidence in the reliable utilization of these threshold values in the response assessment.

To address this issue, some studies have reported promising findings of machine-learning-based radiomic features extracted from anatomical images and diffusion/perfusion MRI-derived images in distinguishing TP from PsP in GBMs.^9,17^ However, most of these studies were focused on using a very large number of shape and texture-based features (overwhelming volume of data), which are often difficult to manage even after applying dimensionality reduction methods, besides being prone to data overfitting and spurious relationships. Moreover, these data-driven radiomics features fail to provide any meaningful physiologically sensitive information and biological interpretation,^18^ which limits the wider adoption of these machine-learning-based prediction models in routine clinical settings.

With these limitations in mind, the present study was conducted with a hypothesis that physiologically sensitive quantitative parameters computed from diffusion tensor imaging (DTI), and dynamic susceptibility contrast (DSC)-perfusion-weighted imaging (PWI) as well as MGMT promoter methylation status combined with machine-learning-based models (a.k.a, radiogenophysiomics) will facilitate distinction of TP from PsP with high accuracy.

## Materials and Methods

**Figure 1.**
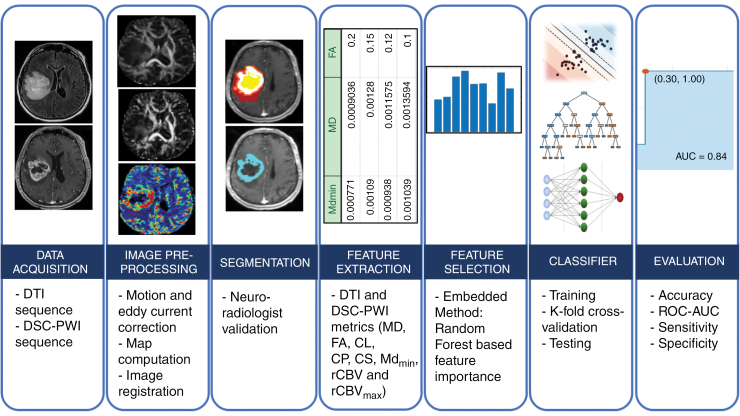
Workflow depicting the steps in developing a prediction model in distinguishing TP from PsP and subsequent evaluation of diagnostic performance by calculating accuracy, sensitivity, specificity, and AUC on a separate, independent test dataset (Abbreviations: DTI, diffusion tensor imaging; DSC-PWI, dynamic susceptibility contrast-perfusion weighted imaging; MD, mean diffusivity; FA, fractional anisotropy; CL, coefficient of linear anisotropy; CP, planar anisotropy; CS, spherical anisotropy; ROC, receiver operating characteristic curve; AUC, area under the ROC curve).

The image processing pipeline as shown in **Figure 1**, provides an overview of data analytical components including image registration, tissue segmentation, feature selection, model-building machine learning-based algorithms, and diagnostic performance metrics.

### Patient Population and Clinical Data

This retrospective study received approval from the institutional review board and adhered to the Health Insurance Portability and Accountability Act. The inclusion criteria for patient enrollment were that all patients: (1) had histologically confirmed diagnosis of GBM, (2) received standard of care (SOC) therapy, ie, surgery and CCRT, (3) exhibited new contrast-enhancing lesion in the radiation field on follow-up MRI within 6 months after completion of SOC therapy, (4) had availability of anatomical and physiological neuroimaging data (DTI, and DSC-PWI). Based upon the inclusion criteria, a cohort of 93 patients (58 males, 35 females; mean age 64.7 ± 8.9 years) was recruited in the present study.

The MGMT promoter methylation status was assessed using a pyrosequencing method on tumor specimens obtained during initial surgery.^19^ Of 93 patients, 75 had the availability of MGMT promoter methylation status. These patients (*n* = 75) were dichotomized into TP (*n* = 55) and PsP (*n* = 20). For 63 patients in whom tumor specimens from repeat surgery/biopsy were accessible, histopathological analyses^20,21^ identified TP (>25% malignant features; *n* = 45) and PsP (<25% malignant features; *n* = 18). In the remaining 12 cases lacking availability of tumor specimens, modified response assessment in neurooncology (mRANO) criteria^22^ were used in establishing TP (*n* = 10) and PsP (*n* = 2) cases by a board-certified neuroradiologist (SM). The tumor size was determined as the sum of the products of diameters (SPD) on the post-contrast T_1_ images. As the mRANO working group has suggested that radiological response at the initial presentation should persist for at least 4 weeks on follow-up imaging before it can be considered as PsP or TP,^22^ tumor size was measured again at the follow-up scan. **A** **Supplementary Figure 1** presents a consort flow diagram illustrating initial patient enrollment, molecular information availability, and subsequent TP/PsP dichotomization.

### Data Acquisition

Patients underwent MRI scans on a 3T MR scanner (Siemens) with a 12-channel phased array head coil. The neuroimaging protocol comprised axial 3D-T_1_-weighted MPRAGE and axial T_2_-FLAIR sequences. Post-contrast T_1_-weighted images were obtained after injecting a gadolinium-based contrast agent using a power injector (Medrad).

### Diffusion Tensor Imaging

DTI data were obtained axially with 30 diffusion directions using a single-shot spin-echo, echo-planar sequence with parallel imaging (acceleration factor of 2). The sequence parameters were as follows: field of view (FOV) = 220 × 220 mm^2^, matrix size = 128 × 128, slice thickness = 3 mm, number of slices = 40, repetition time (TR) = 5,000 ms, echo time (TE) = 86 ms, number of excitations (NEX) = 3, b = 0 and 1000 s/mm^2^. The total acquisition time for the DTI sequence was 8 minutes.

### DSC-Perfusion Weighted Imaging

DSC-PWI data were acquired using a dual bolus injection protocol to mitigate contrast agent leakage effects on CBV measurements. A preloading bolus of 0.07 mmol/kg contrast agent was followed by a second identical bolus during T_2_*-weighted gradient-echo EPI sequence acquisition. The contrast agent was injected at a rate of 5 mL/s followed by 20 mL of saline flush at the same rate. The sequence parameters included: FOV = 220 × 220 mm², matrix size = 128 × 128, slice thickness = 3 mm, TR = 2000 ms, TE = 45 ms, flip angle = 90°, EPI factor = 128, echo spacing = 0.83. Total acquisition time: 3 minutes and 10 seconds. Each slice received 45 sequential measurements with a temporal resolution of 2.17 seconds.

### Image Processing and Data Analysis

The DTI data were preprocessed to minimize motion and eddy current-induced artifacts using an algorithm.^23^ Subsequently, DTI data were analyzed for computing various pixel-wise quantitative parametric maps such as mean diffusivity (MD), fractional anisotropy (FA), coefficient of linear anisotropy (CL), planar anisotropy (CP), and spherical anisotropy (CS) by using the methods reported previously.^23,24^ Pixel-wise leakage-corrected cerebral blood volume (CBV) maps were derived from DSC-PWI data using NordicICE software employing gamma-variate curve fitting for leakage correction.

All quantitative maps and T_2_-FLAIR images were resliced and co-registered to post-contrast T_1_-weighted images. Contrast-enhancing lesions were segmented semiautomatically from post-contrast T_1_-weighted images,^24^ with manual correction by an experienced neuroradiologist (SM) to address the issue of pixel anomalies. Care was taken to avoid large-size normal brain vasculatures from the segmented contrast-enhancing lesions. Median values of DTI metrics and the mean of 10^th^ percentile MD values (MD_min_) were computed from segmented contrast-enhancing regions.^24^ CBV values from enhancing regions were normalized with respect to contralateral normal white matter to obtain relative CBV (rCBV) and the mean of the top 90th percentile rCBV values were reported as rCBV_max_. This method allows reliable estimation of CBV values from the most malignant component of GBMs akin to the “hot-spot” method.^25^

### Quantitative Parameters Dataset Curation

The dataset (dB^fl^) comprised median values of metrics (MD, FA, CL, CP, CS, and rCBV), mean values of 10th percentile MD, mean values of top 90th percentile CBV, and MGMT promoter methylation status from 75 patients, organized into 9 column vectors, each representing a specific feature. The 10th column represented the clinical outcome variable, coded 1 for TP and 0 for PsP. To address the issue of imbalanced sample size between 2 groups of patients (TP [*n* = 55] and PsP [*n* = 20]) that might have caused inaccuracies in the feature selection and radiomics data analysis, a well-established synthetic minority oversampling technique (SMOTE)^26^ was applied to augment the PsP datasets from 20 to 55, resulting in a total sample size of 110. After data balancing, the random shuffling method was used to split the data into 2 subsets in a ratio of 8:2. While 80% of the data (*n* = 90) were used for training and establishing various machine-learning-based prediction models, the remaining 20% of the data (*n* = 20) were used for testing the diagnostic performances of models.

### Feature Importance Computation

The feature importance in the training dataset was ranked using a random forest (RF) algorithm, known for its efficiency in handling correlated and redundant features.^27^ Initially, RF with 100 decision trees was embedded in a loop for binary classification (TP and PsP).^27^ Eventually, 1000 decision trees were chosen, as further increasing the number did not notably improve the classification error.

### Development of Prediction Models

Feature importance estimations and model development were performed using an in-house developed algorithm in MATLAB 2022b platform. After computing feature importance, all features were organized in descending order of significance. Subsequently, a total of 9 widely used machine-learning algorithms including K-nearest neighbors (KNN), logistic regression (LR), support vector machine (SVM) with linear and radial basis function (RBF) kernels, random forest (RF), and various neural network (NN) architectures were employed to construct a robust prediction model for distinguishing TP from PsP. Six-fold cross-validation on dB^fl^ ensured stable results by partitioning the dataset into 6-folds, iteratively training the model on 5 folds, and validating on the remaining fold. This process was repeated 6 times with different validation folds for each model. Initially, the most important feature based on feature importance was selected and used as input for all 9 models. Training and validation accuracy were recorded for each fold and averaged across the 6 folds. In the second step, the second most important feature was selected and combined with the previously selected features as input. The training and validation accuracies were recorded again, and this process was repeated until the feature set was exhausted. The optimal feature set was chosen based on the lowest mean classification error across the 6 validation folds. Parameters and hyperparameters for each classifier were optimized using k-fold (k = 6) cross-validation. For KNN, the optimal k value and Euclidean distance metric were determined. While SVM’s linear kernel (C) and RBF kernel (C and γ) hyperparameters were optimized, RF’s decision tree count and NN’s architecture parameters were fine-tuned accordingly. This comprehensive approach ensured the development of accurate and reliable predictive models for distinguishing TP from PsP cases.

### Statistical Analyses and Performance Evaluation of Prediction Models

Kolmogorov-Smirnov tests were used to determine the nature of MRI data distributions.^28,29^ As the p-values of all parameters were found to be greater than 0.05 using Kolmogorov-Smirnov test, the data were assumed to be normal/Gaussian distributed. Therefore, independent sample T-tests were used to assess differences in the median values of each MRI parameter between TP and PsP groups. Additionally, Pearson’s exact χ^2^ test was performed to estimate the frequency of MGMT promoter methylation in TP and PsP cases. A probability (*P*) value of less than .05 was considered significant.

To evaluate the diagnostic potential of each prediction model in distinguishing TP from PsP, receiver operating characteristic (ROC) curve analyses were performed. The performance metrics included area under the ROC curve (AUC), accuracy, sensitivity, and specificity.

### Determination of Survival Outcomes

Survival analyses were performed to determine the associations between diagnostic groups ((1) TP and PsP and (2) GBMs with MGMT promoter methylation status [methylated and unmethylated]) and overall survival. The survival time of all the patients was recorded from the date of initial diagnosis to the date of death. Patients who were alive at the time of data analysis were censored. Kaplan–Meier survival curve plots and log-rank tests were used to compare the overall survival outcomes in 2 diagnostic groups. Additionally, MRI parameters (median [MD, FA, CL, CP, CS, and rCBV], MD_min_, rCBV_max_) were used as independent stratification factors to determine the overall survival outcomes. Two groups of patients were generated by considering the median values of each MRI parameter (above and below the median). Subsequently, all these variables were incorporated into multivariate survival analysis using the Cox regression hazard model with the backward conditional method. All statistical analyses were performed using SPSS software (v. 18.0).

## Results

**Figure 2.**
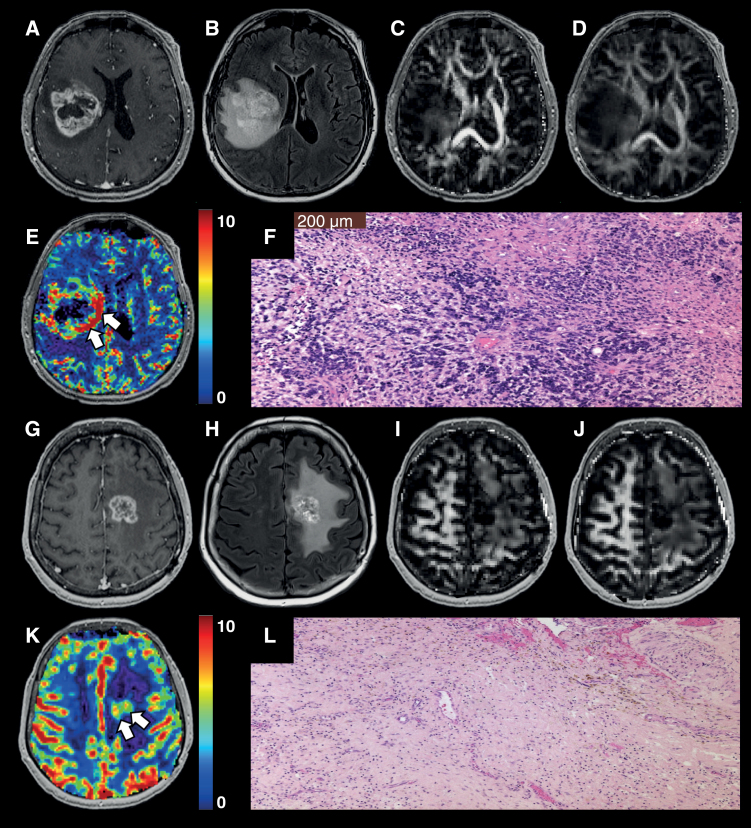
Subfigure(A-F) A 56-year-old male patient with GBM, status post gross total resection and chemoradiation. Post-contrast T_1_-weighted image (A) shows a heterogeneously enhancing lesion located in the right parietal region and extending into the lateral ventricles, which had increased from prior scans. T_2_-FLAIR image (B) demonstrates a large area of associated hyperintense signal abnormality. DTI-derived maps CL (C) and FA (D) show median values of anisotropy indices (CL = 0.04 and FA = 0.12) from the contrast-enhancing regions of the neoplasm. DSC-PWI-derived CBV map (E) shows a markedly elevated rCBV_max_ value of 7.08 from enhancing regions (white arrows). A photomicrograph (F) of hematoxylin-eosin (H & E) stain from this case demonstrates areas of high tumor cellularity, pseudopalisading necrosis, endothelial proliferation, and increased mitotic activity consistent with the findings of true progression (The magnification is 100 × [10× eyepiece, 10× objective lens]). Subfigure(G-L) A 55-year-old male patient with GBM, status post gross total resection and chemoradiation. Post-contrast T_1_-weighted image (G) shows a heterogeneously enhancing lesion at the site of the previously resected neoplasm in the left frontal lobe. T_2_-FLAIR image (H) demonstrates hyperintense signal intensity surrounding the lesion. DTI-derived maps CL (I) and FA (J) show moderate median values of anisotropy indices (CL = 0.04 and FA = 0.10) from contrast-enhancing regions of the neoplasm. DSC-PWI-derived CBV map (K) shows a low rCBV_max_ value of 2.28 from enhancing regions (white arrows). A photomicrograph (L) of hematoxylin-eosin (H & E) stain demonstrates a predominant treatment effect (~80%) with hyalinization of vessels and tissues, geographic necrosis, and chronic lymphocytic infiltration consistent with the findings of PsP. However, infiltrating glial tumor cells with moderate nuclear pleomorphism were also present, comprising approximately 20% of the specimen. (Abbreviations—GBM, glioblastoma; FLAIR, fluid-attenuated inversion recovery; DTI, diffusion tensor imaging; CL, coefficient of linear anisotropy; FA, fractional anisotropy; DSC-PWI, dynamic susceptibility contrast-perfusion weighted imaging; CBV, cerebral blood volume; rCBV, relative cerebral blood volume; TP, true progression; PsP, pseudoprogression).

**Figure 2** shows representative anatomical MR images, quantitative DTI maps (CL, FA), and CBV maps from patients with TP and PsP, respectively. The distributions of DTI and DSC-PWI-derived parameters from contrast-enhancing regions of neoplasms from all patients are shown as box-and-whisker plots (**Supplementary Figure 2**).

Significantly higher rCBV_max_ (3.41 ± 1.42 vs. 2.76 ± 0.89, *P* = .023), rCBV (1.77 ± 0.80 vs. 1.46 ± 0.44, *P* = .038), FA (0.14 ± 0.05 vs. 0.11 ± 0.02, *P* = .032) and CP (0.07 ± 0.02 vs. 0.06 ± 0.01, *P* = .047) values were observed from TP than those from PsP cases. There were no significant differences in other imaging parameters between the 2 groups of patients (*P* > .05). The χ^2^ test revealed that the frequency of GBMs harboring unmethylated MGMT promoter status was significantly higher in TP (*n* = 45/55) than in PsP (*n* = 8/20; *P* < .01, **Supplementary Figure 3****).**

**Figure 3.**
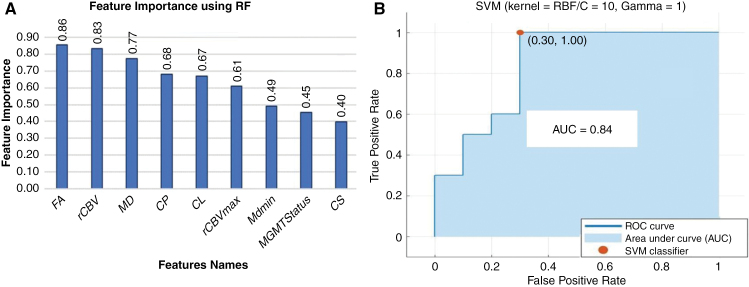
The bar plots (A) represent the feature importance (in descending order) based on a random-forest algorithm. The ROC curve (B) exhibits an AUC of 0.84 for the best prediction model (SVM with RBF kernel) in distinguishing TP from PsP. (Abbreviations- FA, fractional anisotropy; rCBV, relative cerebral blood volume; MD, mean diffusivity; CL, coefficient of linear anisotropy; CP, planar anisotropy; CS, spherical anisotropy; ROC, receiver operating characteristic curve; AUC, area under the ROC curve; SVM, support vector machine).

**Figure 4.**
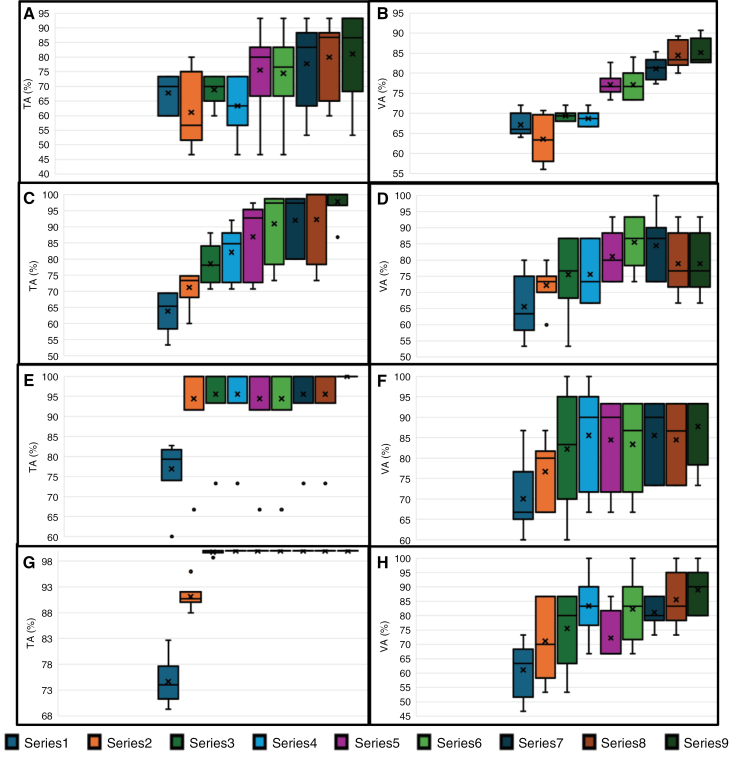
The Boxplot labeled A to H depicts the classification training and cross-validation accuracies of logistic regression (LR), support vector machine with a radial basis kernel (quadratic SVM), random forest (RF), medium neural network (MNN) across the 6-folds, with Series 1 to Series 9 representing feature vectors. Feature vector: Series1={FA}, Series2= {FA, rCBV}, Series3={FA, rCBV, MD}, Series4= {FA, rCBV, MD, CP}, Series5= {FA, rCBV, MD, CP, CL}, Series6= {FA, rCBV, MD, CP, CL, rCBV_max_}, Series7= {FA, rCBV, MD, CP, CL, rCBV_max_, MD_min_}, Series8= {FA, rCBV, MD, CP, CL, rCBV_max_, MD_min_, MGMT}, Series9= {FA, rCBV, MD, CP, CL, rCBV_max_, MD_min_, MGMT, CS}. (Abbreviations- FA, fractional anisotropy; rCBV, relative cerebral blood volume; MD, mean diffusivity; CL, coefficient of linear anisotropy; CP, planar anisotropy; MGMT, O^6^-methylguanine-DNA-methyltransferase; CS, spherical anisotropy).

The RF-driven feature importance plot is shown in **Figure 3A**. Among all the independent features, FA, rCBV, and MD were identified as the highest-ranked predictors, while MGMT promoter methylation status and CS were ranked lower in distinguishing TP from PsP. **Figure 4** presents boxplots (A-H) showing classification training and cross-validation accuracies for LR, SVM, RF, and medium neural networks across 6-folds, with Series1 to Series9 representing feature vectors arranged in the descending order of feature importance. As shown in **Table 1**, the RF and NN (narrow, mediumNN, bilayeredNN, trilayeredNN) machine-learning-based prediction models resulted in 100% training accuracies. However, the mean 6-fold cross-validation accuracies were 88%, 88%, 89%, 82%, and 88%, respectively. On the other hand, the training accuracies for KNN, LR, and SVMs (linear and RBF kernels) machine-learning-based prediction models were 79%, 85%, 80%, and 91%, and the respective cross-validation accuracies were 76%, 81%, 79%, and 86%, respectively.

**Table 1.** The Training Accuracies(TA), 6-Fold Cross-Validation Accuracies(VA) and Testing Accuracies(TE), along With Corresponding Sensitivities and Specificities for Different Machine Learning Based Prediction Models in Distinguishing TP and PsP are Presented

Classification algorithm	Optimized features	Optimized hyperparameters	TA(%)	VA (%)	TE(%)	Sensitivity	Specificity
KNN (NN = 10)	FA, rCBV, MD, CP, CL, rCBV_max_, Mdmin, MGMT,CS	NN = 10	79	76	80	70	90
LR	FA, rCBV, MD,CP,CL, rCBV_max_, Mdmin, MGMT,CS		85	81	70	90	50
Linear SVM	FA, rCBV, MD,CP,CL, rCBV_max_, Mdmin, MGMT	C = 1	80	79	60	70	50
Quadratic SVM	FA, rCBV, MD, CP, CL, rCBV_max_	C = 10, Gamma = 1	91	86	85	70	100
Ensemble Learning(RF)	FA, rCBV, MD,CP,CL, rCBV_max_, Mdmin, MGMT,CS	No. of learners = 100 / No. of split = 74(Number of samples in the considered dataset)	100	88	85	70	100
NN	FA, rCBV, MD,CP,CL, rCBV_max_, Mdmin, MGMT,CS	Number of fully connected layers: 1, First layer size: 10, Activation: ReLU Iteration limit: 1000	100	88	85	60	100
MNN	FA, rCBV, MD,CP,CL, rCBV_max_, Mdmin, MGMT,CS	Number of fully connected layers: 1, First layer size: 25, Activation: ReLU Iteration limit: 1000	100	89	85	70	100
bi-NN	FA, rCBV, MD,CP, CL, rCBV_max_	Number of fully connected layers: 2, First layer size: 10, Second layer size: 10, Activation: ReLU, Iteration limit: 1000	100	82	80	60	100
tri-NN	FA, rCBV, MD, CP, CL, rCBV_max_	First layer size: 10, Second layer size: 10, Third layer size: 10, Activation: ReLU, Iteration limit: 1000	100	88	80	70	100

Abbreviations: KNN, K-nearest neighbors; LR, logistic regression; SVM, support vector machine; RF, random forest; NNN, narrow neural network; MNN, medium neural network; Bi-layered NN, Bi-layered neural network; Tri-layered NN, Tri-layered neural network; MD, mean diffusivity; FA, fractional anisotropy; CL, coefficient of linear anisotropy; CP, planar anisotropy; CS, spherical anisotropy; CBV, cerebral blood volume; rCBV, relative cerebral blood volume; MGMT, O^6^-methylguanine-DNA-methyltransferase.

Finally, the SVM with RBF kernel (optimized hyperparameters: C = 10/ γ = 1) algorithm developed using the optimized feature set including FA, rCBV, MD, CP, CL, and rCBV_max_, was chosen for the final model development. This algorithm emerged as the most efficient machine-learning model among all implemented algorithms in terms of overfitting, bias, and variance. SVM (RBF kernel) based prediction model provided a testing accuracy of 85%, the sensitivity of 70%, and specificity of 100% for distinguishing TP from PsP. Additionally, ROC analysis revealed an AUC of 0.84 in distinguishing TP from PsP (**Figure 3B**).

**Figure 5.**
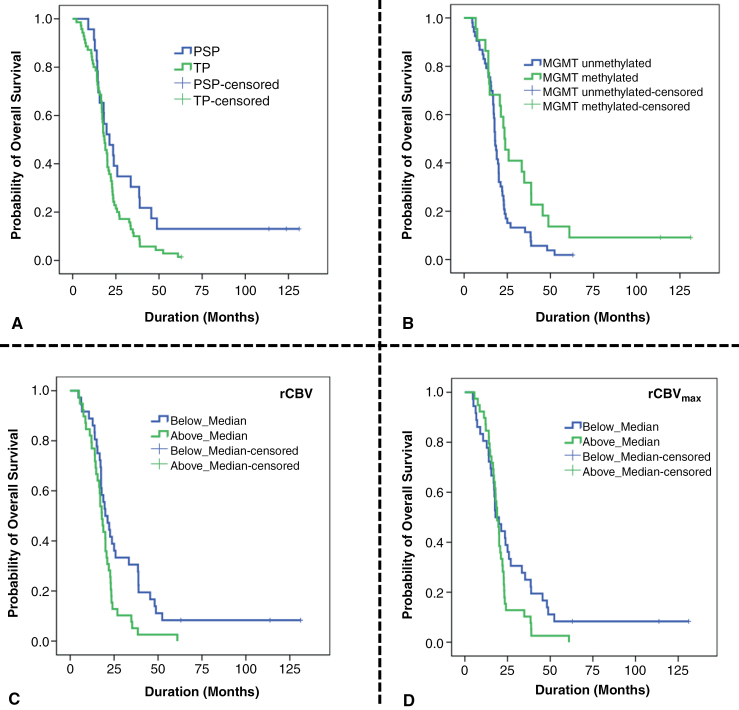
Kaplan–Meier (KM) curves illustrate that patients with PsP have significantly longer overall survival compared to those with TP (log-rank *P* = .043) in subfigure (A). Additionally, GBM patients with MGMT promoter methylation status exhibit longer overall survival than those without (log-rank *P* = .015) in subfigure (B). KM curves also show that GBM patients with lower median rCBV have significantly longer survival times compared to those with higher median rCBV (20.5 ± 2.47 vs. 18.1 ± 1.09 months, log-rank *P* = .011) in subfigure (C). Furthermore, there is a trend towards longer survival for GBM patients with lower rCBV_max_ (17.9 ± 2.4 vs. 18.9 ± 1.24 months, log-rank *P* = .081) in subfigure (D) (Abbreviations–PsP, pseudoprogression; TP, true progression; GBM, glioblastoma; MGMT, O^6^-methylguanine-DNA-methyltransferase; rCBV, relative cerebral blood volume).

The median overall survival from the date of initial diagnosis to the date of death for all patients was 18.9 months. Kaplan–Meier survival analyses revealed that PsP patients had significantly prolonged overall survival (21.3 ± 4.3 vs. 18.3 ± 1.1 months, log-rank *P* = .043) than TP patients (**Figure 5).** When survival analyses were performed using MGMT promoter methylation status as an independent variable, MGMT methylated GBM patients had significantly longer survival than those with unmethylated MGMT promoter (23.4 ± 2.6 vs. 17.9 ± 0.6 months, log-rank *P* = .015). Using MRI parameters as independent stratifying factors, Kaplan–Meier survival curves demonstrated that GBM patients with lower rCBV had significantly longer survival time than those with higher median rCBV (20.5 ± 2.47 vs. 18.1 ± 1.09 months, log-rank *P* = .011). Additionally, a trend towards significant survival time was obtained for GBM patients with lower rCBV_max_ (17.9 ± 2.4 vs. 18.9 ± 1.24 months, log-rank *P* = 0.081). Multivariate Cox-regression analyses revealed that MGMT status (hazard ratio = .573, 95% CI: 0.33–0.98; *P* = .044) and rCBV (hazard ratio = 1.71, 95% CI: 1.04–2.81; *P* = .035) were still identified as significant prognostic factors in determining survival outcomes even after adjusting age and gender as confounding factors.

## Discussion

There is an unmet need to develop a prediction model for providing accurate and early characterization of a lesion as TP or PsP following surgery and CCRT in GBM patients. This has great potential for prognostication and making go-stop decisions on future therapeutic interventions in these patients. While PsP patients may continue standard therapy, TP patients may benefit from the early institution of second-line treatment including repeat surgery or alternative therapies. In the present study, we sought to develop a machine-learning model using MRI and molecular data to differentiate TP from PsP in GBM patients. Our findings suggest an automated SVM model comprising DTI and DSC-PWI-derived parameters from contrast-enhancing regions of neoplasms can achieve high diagnostic performance (85% accuracy, 70% sensitivity, and 100% specificity, respectively).

Using quantitative metrics derived from DTI and DSC-PWI, previous studies have reported sensitivities and specificities in the range of 62%–91% in distinguishing TP from PsP.^13,30,31^ The large variations in the reported values may be attributed to the fact that DTI and DSC-PWI sequences were used independently in most of those studies. Moreover, imaging parameters of DTI and DSC-PWI sequences were not integrated to obtain a reliable classification model. In a previous study^31^ multivariate LR method was used to develop a prediction model by combining together quantitative parameters derived from DTI and DSC-PWI sequences in the classification of TP and PsP cases with 57%–90% accuracy. Moving forward, the diagnostic performance of this multiparametric MRI-based prediction model was validated in a new independent cohort of GBM patients receiving CCRT with an accuracy of 76%.^21^ LR is a traditional statistical method that is commonly employed to develop prediction models for examining associations between clinical outcomes and independent variables.^31^ Despite being associated with high calibration capability, interpretability, and ease of implementation, LR methods sometimes fail to capture complex, nonlinear relationships between the predictors and clinical outcomes.^32^ While some studies have indicated that LR and other machine-learning-based prediction models demonstrate comparable overall performance in clinical applications,^33^ some other studies have documented that advanced machine-learning models possess superior prediction capabilities compared to LR-based prediction models.^34^ Furthermore, advanced machine-learning classifiers exhibit a greater ability to effectively handle missing data compared to LR-based models.^34^

Lately, several studies have reported the potential utility of machine-learning-based algorithms in discriminating TP from PsP in GBMs.^9,11,21,30,35^ However, most of those studies used high-dimensional shape/texture features such as gray-level co-occurrence matrix (GLCM), gray-level co-dependence matrix (GLDM), and gray-level run-length matrix extracted from anatomical and /or physiological sensitive images (eg, MD, CBV maps). These shape/texture-based features are highly sensitive to variations in image acquisition and processing protocols and are not associated with clinically meaningful interpretations related to tumor microenvironment and biology. In contrast, DTI and DSC-PWI-derived parameters (eg, MD, FA, CL, and CBV) are widely used in clinical practice and directly provide physiological linkage to the tumor microenvironment and biology.^8^ In the present study, the RF algorithm was used to determine the relative importance of features. Among all independent features, FA, rCBV, and MD were identified as the highest-ranked predictive parameters for distinguishing TP from PsP. On DTI, successful treatment is exhibited by an increase in MD from solid/contrast-enhanced regions of neoplasms, reflecting the destruction of tumor cells and reduction of barriers to proton motion.^31,36,37^ Additionally, reduced FA has been associated with positive treatment outcomes secondary to reduced cell density and/or incoherent orientation of neoplastic cells following treatment.^38^ Neovascularization is a common feature of GBMs that accounts for high tumor perfusion, as seen on CBV maps.^39^ Several studies have reported reduced rCBV in GBMs following CCRT, anti-angiogenic therapy, and immunotherapy suggesting the potential utility of rCBV in evaluating treatment response.^15,31,40^ In a study by Liu et al. fibrinoid necrosis, endothelial injury, and occlusion of blood vessels have been proposed as potential reasons for decreased rCBV in treated brain tumors.^41^ In the current study, reduced values of rCBV and rCBV_max_ were noted in PsP compared to those in TP cases, and these findings were concordant with previously reported studies.^15,31,40^ When MRI parameters were used to determine the survival outcomes, median rCBV was found to be a significant predictor of overall survival. Additionally, a trend towards significance was observed for rCBV_max_ in determining the overall survival in our patient population. In particular, GBM patients harboring lower values of rCBV had prolonged overall survival than those with higher rCBV. A previous study also reported that pretreatment rCBV_max_ may be used as a sensitive prognostic marker for overall survival in GBM patients.^42^ The reduced rCBV values suggest a decrease in vascularity and perfusion within the tumor beds in GBMs and better clinical outcomes in these patients.

The current study employed 9 commonly used machine-learning classifiers on 9 radiomics features, including 6 from DTI (MD, FA, CL, CP, CS, and MD_min_), 2 from DSC-PWI (rCBV and rCBV_max_), and one molecular signature (MGMT promoter methylation status) to construct prediction models. Classifiers were selected based on accuracy, training duration, handling of missing data, and ease of interpretation. ROC curve analyses indicated that RF and various NN classifiers outperformed others in discriminatory accuracy, however, these classifiers were prone to data overfitting behavior. On the other hand, the optimized SVM classifier with RBF kernel function provided improved performance in terms of bias and variance and was considered to be better equipped to handle the data. SVM is a robust binary classification technique, which has shown great potential for distinguishing TP from PsP in previous studies.^43,44^

Another key finding in the present study was that SVM (RBF kernel) classifier selected multiple parameters (MD, FA, CL, CP, rCBV, and rCBV_max_) in the model development and yielded high discriminatory accuracies from training (91%), testing (85%), and cross-validation (85%) data sets in distinguishing TP from PsP. A multiparametric approach exploiting the unique strengths of different diagnostic techniques allows a comprehensive assessment of complex tumor biology and microenvironment. DTI and DSC-PWI parameters offer inherently different but complementary physiological information, potentially synergizing in combined analysis to improve diagnostic accuracy beyond individual parameters. Studies have shown that a multiparametric radiomics model outperforms monoparametric models, suggesting its superiority in tumor characterization.^17,45^ Similarly, a combination of 6 parameters selected in the SVM-based model provided the best accuracy in distinguishing TP and PsP in the present study. Collectively, these findings further emphasize the significance of utilizing a multiparametric approach even if we use more sophisticated machine-learning-based models in the evaluation of treatment response in GBMs.

In our study, PsP patients showed significantly prolonged overall survival compared to TP patients. Additionally, patients showed longer overall survival in the MGMT methylated than in the MGMT unmethylated group. Previous studies have reported that GBM patients harboring methylated MGMT promotor treated with SOC treatment are associated with favorable clinical outcomes and greater response to treatment than those harboring unmethylated genotype.^46,47^ MGMT promoter methylation status is considered an independent prognostic factor in GBM patients and it is widely believed that PsP harbor improved survival outcomes in part due to its association with methylated MGMT promoter status.^46,47^ Despite the presence of significantly higher frequency of MGMT promotor methylation profile in PsP than in TP groups in the present study, MGMT promotor methylation status was not selected as a relevant feature in SVM (RBF kernel) based prediction model. Though this observation was unexpected, a similar finding has also been reported previously.^17^ On the contrary, some studies have reported improved diagnostic performance of radiomics-based models when MGMT promotor methylation data were incorporated into the model development.^45,48^ Probably, these contradictory findings might have arisen due to the utilization of different machine learning-based classifiers in those studies. However, non-selection of molecular information in the prediction model may actually be beneficial in some settings especially in resource-limited clinical environments when it is not usually possible to obtain MGMT promoter methylation status in GBMs.

For tumor specimens, which present with predominant malignant features or predominant treatment-related changes, diagnosis is relatively straightforward. However, most of the tumor specimens consist a combination of viable tumor cells and treatment-related changes. These patients present with mixed treatment response,^49^ and are closely monitored with short-interval follow-up MRI scans and subjected to repeat surgery and/or to alternate therapies in a similar manner as those with TP in the usual clinical practice.^43,50^ Therefore, keeping the best clinical practice in mind and for appropriate treatment stratification, patients in the present study (in whom tumor specimens from repeat surgery/biopsy were accessible) were dichotomized into 2 groups TP (>25% malignant features) and PsP (<25% malignant features) based on histopathological analyses.

While presenting promising findings, our study had some shortcomings including the retrospective nature of study design. Additionally, 12/75 patients did not undergo repeat surgery/biopsy, and we therefore did not have histopathological determination for a “ground truth” diagnosis of TP and PsP in these cases. Nonetheless, we employed well-established mRANO criteria of using more than 2 consecutive conventional neuroimaging findings to ascertain TP or PsP status in these cases. Although we did not have any selection bias for recruiting patients in the present study, the ratio of TP to PsP patients was almost 3:1, which is in line with the reported incidence rates of PsP in all GBM patients undergoing CCRT.^2^ Another limitation was related to imbalanced dataset that might lead to model over-fitting behavior towards the majority class (TP). In order to address this issue, the SMOTE algorithm (a data resampling method) was used to artificially augment the data size of minority class (PsP cases) in the present study. Additionally, clinical variables such as Karnofsky performance status score, tumor location, tumor size, the extent of tumor resection, etc. were not considered while developing machine learning-based prediction models in the present study. Besides quantitative MRI parameters, the integration of clinical variables may further improve the diagnostic accuracy of prediction models in future studies.

In the present study, all the patients underwent MRI with identical protocol and sequence parameters. However, it is well known that even small differences in hardware/software components or sequence parameters may result in substantial variations in image signal intensity and tissue contrast patterns, rendering the interpretation of imaging results obtained from different MRI protocols, scanner vendors, and treatment centers complicated and hence inconsequential. Therefore, widespread adoption of these imaging techniques into routine clinical workflow requires standardization, harmonization of data acquisition, and processing protocols, along with the application of well-defined quality assessment/control procedures. Fortunately, consensus guidelines have been proposed to implement MR diffusion, and perfusion imaging techniques across different clinical sites to enhance the reproducibility and reliability of physiologic MRI techniques.^51,52^ Additional improvements in this field require data sharing, data federation, and conduction of large multicentric validation studies.

## Conclusion

In conclusion, the SVM model comprising physiologically sensitive and quantitative MRI parameters may provide an accurate, objective, and therapy-agnostic machine learning-based tool in distinguishing TP from PsP in GBMs treated with standard therapy *(precision diagnostics)*. An early window of opportunity will enable optimal and timely therapeutic interventions in these patients *(personalized therapeutics)*. However, future large-scale multi-site studies are required to confirm our findings.

## Supplementary Material

vdae159_suppl_Supplementary_Figures_S1-S3
