# Supplementary material for: Distinction of pseudoprogression from true progression in glioblastomas using machine learning based on multiparametric magnetic resonance imaging and O6-methylguanine-methyltransferase promoter methylation status
Source: Neurooncol Adv. 2024 Oct 3;6(1):vdae159. doi: 10.1093/noajnl/vdae159 (PMC11535496; doi:10.1093/noajnl/vdae159)
Supplement: vdae159_suppl_Supplementary_Figures_S1-S3 [file vdae159_suppl_Supplementary_Figures_S1-S3.zip › Supplementary Figure Legends.docx]

**Supplementary Figure 1.** Flow diagram showing the patient selection protocol for final data analysis.

**Supplementary Figure 2.** Boxplots of diffusion (FA, CP, MD, Md_min_, CL, CS) and perfusion (rCBV and rCBV_max_ ) MRI characteristics for patients with TP (gray), and PsP (white). The solid line inside each box represents the median value, while the edges represent the 25^th^ and 75^th^ percentiles. The straight line (bars) on each box indicates the range of data distribution. The circle represents the outlier (values 1.5 box length from the 75^th^ /25^th^ percentiles).

**Supplementary Figure 3.** Bar plots showing the frequency of GBMs harbouring MGMT promotor methylation status in PsP and TP patients.
